# Clinical, Metabolic and Hormonal Overlap Between Nonclassic Congenital Adrenal Hyperplasia and Polycystic Ovary Syndrome: An Exploratory Study

**DOI:** 10.3390/jcm15176522

**Published:** 2026-08-23

**Authors:** Arzu Yavuz, Aylin Coskun, Dilara Tekin Uzman, Esra Hatipoglu

**Affiliations:** 1 Department of Obstetrics and Gynecology, Kocaeli City Hospital, University of Health Sciences, Kocaeli 41060, Türkiye; 2 Division of Endocrinology, Department of Internal Medicine, Basaksehir Cam and Sakura City Hospital, University of Health Sciences, Istanbul 34480, Türkiye; aylinciftkaya@gmail.com (A.C.); dresrah@gmail.com (E.H.)

**Keywords:** polycystic ovary syndrome, nonclassic congenital adrenal hyperplasia, 17-hydroxyprogesterone, hyperandrogenism, Rotterdam phenotypes, anti-Müllerian hormone

## Abstract

**Objective:** Polycystic ovary syndrome (PCOS) and nonclassic congenital adrenal hyperplasia (NCAH) overlap clinically, but direct comparative data specifically addressing non-National Institutes of Health (non-NIH) Rotterdam phenotypes of polycystic ovary syndrome are limited. We compared women with polycystic ovary syndrome, of whom 66.7% were classified as having non-NIH Rotterdam phenotypes, with women with nonclassic congenital adrenal hyperplasia and healthy controls to characterize the extent of phenotypic overlap and evaluate the discriminatory performance of individual androgen measures. **Methods:** This single-center, prospective, exploratory cross-sectional study evaluated 58 women divided into three groups: PCOS (*n* = 24, Rotterdam criteria), NCAH due to 21-hydroxylase deficiency (*n* = 16, previously confirmed by adrenocorticotropic hormone (ACTH)-stimulated 17-hydroxyprogesterone (17-OHP)), and healthy controls (*n* = 18). Demographic, clinical, metabolic, and hormonal parameters were compared using analysis of variance (ANOVA), Kruskal–Wallis with Dunn’s post hoc test (Bonferroni-adjusted), and chi-square or Fisher’s exact tests. A sensitivity analysis was performed after excluding 12 participants receiving oral contraceptives (*n* = 8) or glucocorticoids (*n* = 4). **Results:** Within the PCOS group, 66.7% had non-NIH Rotterdam phenotypes. Hirsutism, defined as a modified Ferriman–Gallwey (mFG) score ≥ 8, was more frequent in both patient groups than in controls (*p* = 0.003) but did not differ between PCOS and NCAH. Total testosterone levels were 0.40 [0.20–0.60], 0.59 [0.45–0.94], and 0.20 [0.15–0.20] ng/mL in the PCOS, NCAH, and control groups, respectively, and androstenedione levels were 1.40 [1.20–2.20], 2.55 [1.64–4.07], and 0.87 [0.56–1.00] ng/mL, respectively; both were higher in the patient groups than in controls (both *p* < 0.001), without differing between PCOS and NCAH. Basal 17-OHP was the only parameter distinguishing NCAH from both PCOS and controls (*p* < 0.001). Dehydroepiandrosterone sulfate (DHEAS) levels were 356.5 [217–422], 292 [156–448], and 219 [146–240] µg/dL, respectively (*p* = 0.020), with a significant difference only between PCOS and controls. Anti-Müllerian hormone (AMH) levels were 3.27 [3.02–4.13], 3.13 [2.87–3.54], and 3.34 [2.93–5.37] ng/mL, respectively, with no between-group difference (*p* = 0.600). Other metabolic parameters, gonadotropins, estradiol, and prolactin showed no between-group differences. The principal hormonal findings remained unchanged after excluding women receiving oral contraceptives or glucocorticoids. **Conclusions:** In this PCOS cohort, in which 66.7% of women were classified as having non-NIH Rotterdam phenotypes, PCOS and NCAH showed substantial clinical, metabolic, and hormonal overlap, whereas basal early-follicular 17-OHP was the only parameter that consistently distinguished NCAH from both PCOS and healthy controls. These findings support the inclusion of basal 17-OHP in the evaluation of women presenting with hyperandrogenism.

## 1. Introduction

Polycystic ovary syndrome (PCOS) is the most common endocrine disorder in women of reproductive age, affecting approximately 10–13% of this population, and is characterized by hyperandrogenism, ovulatory dysfunction, and polycystic ovarian morphology (PCOM) [1,2,3]. Under the 2003 Rotterdam criteria, diagnosis requires at least two of these three features, generating four recognized phenotypes: A (hyperandrogenism + ovulatory dysfunction + PCOM), B (hyperandrogenism + ovulatory dysfunction), C (hyperandrogenism + PCOM), and D (ovulatory dysfunction + PCOM) [4]. Phenotypes A and B, which both include hyperandrogenism and ovulatory dysfunction, correspond to the older 1990 National Institutes of Health (NIH) definition and are frequently termed classic or NIH PCOS, whereas C and D constitute the non-NIH Rotterdam phenotypes [4]. This phenotypic heterogeneity is clinically consequential, as anti-Müllerian hormone (AMH) concentrations, androgen levels, and metabolic burden differ substantially across phenotypes, with the non-NIH groups, particularly the ovulatory, hyperandrogenic phenotype C, representing the milder end of the spectrum [5,6,7].

Nonclassic congenital adrenal hyperplasia (NCAH), most commonly due to partial 21-hydroxylase deficiency, is an autosomal recessive disorder in which residual enzyme activity leads to an accumulation of 17-hydroxyprogesterone (17-OHP) and a shift toward adrenal androgen production [8,9]. The prevalence of NCAH is estimated at approximately 0.1–1% in the general population, with substantial variation according to ethnicity [8]. Unlike PCOS, which arises from a heterogeneous, multifactorial pathogenesis, NCAH represents a defined monogenic enzymatic defect. Nevertheless, its clinical presentation in adolescent and adult women is dominated by androgen excess, hirsutism, acne, and menstrual irregularity, rather than adrenal insufficiency, and therefore closely mimics PCOS [8,9,10,11]. Accurate distinction between PCOS and NCAH is essential because their management may differ, and, unlike PCOS, NCAH is an inherited autosomal recessive disorder with implications for genetic counseling and reproductive planning [8,12,13].

The clinical overlap between PCOS and NCAH is substantial. In a systematic review, hirsutism was reported in 59% of women with NCAH and 60–70% of women with PCOS, whereas acne was reported in 33% and 14–25%, respectively. Menstrual irregularity (17% vs. 90%) and infertility (13% vs. 25–50%) were less frequently reported in NCAH than in PCOS [9]. Both disorders are associated with obesity, insulin resistance, and dyslipidemia, so clinical features alone are generally insufficient for the differential diagnosis [9,14]. PCOM, although more common in PCOS, also occurs in NCAH and therefore cannot by itself rule the diagnosis in or out [8,11]. Circulating androgens, including androstenedione, testosterone, and sometimes DHEAS, tend to be elevated in both disorders and overlap considerably, yet none is as specific as 17-OHP [11,15]. The interpretation of androgen concentrations is also influenced by assay methodology, particularly at the low circulating concentrations observed in women, where direct immunoassays have limited sensitivity and precision compared with liquid chromatography–tandem mass spectrometry (LC-MS/MS) [3]. Consequently, the prevailing evidence-based position is that PCOS and NCAH cannot be reliably separated by symptoms, ultrasound, or metabolic features alone, and the differential diagnosis rests on basal early-follicular 17-OHP as the principal screening test, followed by ACTH (cosyntropin) stimulation when basal values are borderline [8,11].

Despite this well-established framework, direct comparative data specifically addressing non-NIH Rotterdam phenotypes of PCOS in relation to NCAH remain limited. Previous comparative studies have generally evaluated PCOS as a broader diagnostic group rather than specifically focusing on these milder phenotypes [9,11]. As a result, the specific comparison between a PCOS cohort in which 66.7% of women had non-NIH phenotypes and NCAH has received little direct attention. This is clinically meaningful because the non-NIH phenotypes, metabolically mild yet hyperandrogenic, plausibly represent the PCOS subgroup most likely to be confused with NCAH and the group in which reassuringly normal metabolic and ovarian-reserve findings might falsely lower clinical suspicion of an underlying enzymatic defect. Given the emerging role of AMH in the diagnostic evaluation of PCOS and its variation across PCOS phenotypes, we also investigated whether AMH could provide additional discriminatory information in differentiating PCOS from NCAH. We therefore aimed to compare a PCOS cohort in which 66.7% of women had non-NIH phenotypes with women who had NCAH and healthy controls across demographic, anthropometric, clinical, metabolic, and hormonal parameters in order to characterize the degree of phenotypic overlap and evaluate the discriminatory performance of individual androgen and steroid measures, including 17-OHP, in this diagnostically challenging setting. We hypothesized that PCOS and NCAH would show substantial overlap in clinical and conventional androgen parameters, whereas basal 17-OHP would provide the clearest biochemical discrimination between the two conditions; we further hypothesized that AMH might provide additional discriminatory information.

## 2. Materials and Methods

### 2.1. Study Design and Population

This single-center, prospective, exploratory, cross-sectional study was conducted at Basaksehir Cam and Sakura City Hospital, University of Health Sciences, Istanbul, Turkiye. The study protocol was approved by the Basakşehir Cam and Sakura City Hospital Clinical Research Ethics Committee (approval number E-96317027-514.10-292447764, dated 27 October 2025) and was conducted in accordance with the principles of the Declaration of Helsinki. Written informed consent was obtained from all participants.

Between December 2025 and June 2026, all eligible women of reproductive age with previously established diagnoses who attended our endocrinology outpatient clinic during the study period were consecutively invited to participate. Those who agreed to participate and met the study criteria were enrolled. Three groups were formed: women with polycystic ovary syndrome (PCOS), women with nonclassic congenital adrenal hyperplasia (NCAH) due to 21-hydroxylase deficiency, and healthy controls. Women in the NCAH group had a previously established diagnosis of NCAH and were under regular follow-up for this condition at our endocrinology outpatient clinic; they were not identified through screening of patients initially diagnosed with PCOS. Healthy controls were volunteers who attended the endocrinology outpatient clinic for reasons unrelated to hyperandrogenism and who met the predefined criteria for the control group described below.

### 2.2. Diagnostic Criteria and Group Definitions

PCOS was diagnosed according to the 2003 Rotterdam criteria, requiring at least two of the following three features after exclusion of other causes of hyperandrogenism: clinical and/or biochemical hyperandrogenism, oligo-/anovulation, and polycystic ovarian morphology (PCOM). In accordance with the 2023 International Evidence-Based Guideline, PCOS was defined as the presence of ≥20 follicles per ovary and/or an ovarian volume ≥ 10 mL. In the present cohort, PCOM was based on the ovarian morphology described in ultrasonography reports; however, systematic follicle counts and ovarian volume measurements were not consistently available, and this criterion could therefore not be uniformly applied across all participants.

NCAH due to 21-hydroxylase deficiency had been previously diagnosed in these patients on the basis of an ACTH (cosyntropin)-stimulated 17-hydroxyprogesterone (17-OHP) level > 10 ng/mL, following a basal early-follicular 17-OHP level above 2 ng/mL that prompted stimulation testing. For the present analysis, basal early-follicular 17-OHP levels were used for between-group comparisons.

Healthy controls were women with regular menstrual cycles, defined as cycle lengths of 21–35 days and without clinical or biochemical evidence of hyperandrogenism. For all groups, exclusion criteria were pregnancy; thyroid dysfunction; hyperprolactinemia, including prolactinoma; Cushing’s syndrome; and androgen-secreting adrenal or ovarian tumors.

### 2.3. Data Collection and Measurements

Demographic, anthropometric, clinical, metabolic, and hormonal data were recorded. Anthropometric assessment included body mass index (BMI). Hirsutism was defined as a modified Ferriman–Gallwey (mFG) score of ≥8, based on available clinical assessments performed by an experienced endocrinologist. The mFG score was also analyzed as a continuous variable. Reproductive history, including the number of healthy pregnancies, parity, and abortions, was also recorded.

Blood samples for hormonal and metabolic parameters were obtained in the early follicular phase (days 2–5) of the menstrual cycle. In women with oligo-/amenorrhea, samples were collected in the early follicular phase following progestin-induced withdrawal bleeding. No mandatory washout period for oral contraceptives or glucocorticoids was required before baseline blood sampling; participants receiving these medications at the time of evaluation were included in the primary analysis. To assess the potential influence of these treatments, a sensitivity analysis was subsequently performed after excluding participants receiving oral contraceptives or glucocorticoids. Hormonal parameters included follicle-stimulating hormone (FSH), luteinizing hormone (LH), estradiol (E2), total testosterone, androstenedione, dehydroepiandrosterone sulfate (DHEAS), 17-OHP, prolactin, and anti-Müllerian hormone (AMH). Metabolic parameters included fasting glucose, insulin (with calculation of the homeostatic model assessment of insulin resistance [HOMA-IR]), glycated hemoglobin (HbA1c), and a lipid panel comprising triglycerides, high-density lipoprotein (HDL) cholesterol and low-density lipoprotein (LDL) cholesterol.

FSH, LH, estradiol, total testosterone, prolactin, DHEAS, and insulin were measured by electrochemiluminescence immunoassay (ECLIA) on the Cobas e801 Analytical Unit (Roche Diagnostics, Mannheim, Germany). Serum 17-OHP was analyzed by immunoassay using the DiaMetra 17OH Progesterone ELISA kit (DiaMetra Srl Unipersonale, Spello, Italy) on an Alisei Q.S. ELISA Analyzer (SEAC, Calenzano, Italy).

Androstenedione was measured by liquid chromatography–tandem mass spectrometry (LC-MS/MS). Serum AMH was measured by a sandwich enzyme-linked immunosorbent assay (Human MIS/AMH ELISA Kit, Cat. No. E1052Hu; Bioassay Technology Laboratory, Jiaxing, Zhejiang, China), with a detection range of 0.05–15 ng/mL, sensitivity of 0.01 ng/mL, and intra- and inter-assay coefficients of variation below 8% and 10%, respectively. Fasting glucose was determined by the hexokinase method and HbA1c by HPLC (MQ 8000 PT, Medconn Diagnostics, Shanghai, China). Lipid parameters, including triglycerides, HDL-C, and LDL-C, were assessed using colorimetric and homogeneous enzymatic assays (Trigl, HDLC4, and LDLC3, respectively; Roche Diagnostics, Mannheim, Germany) on the Cobas c702 system. HOMA-IR was calculated as fasting insulin (µIU/mL) × fasting glucose (mg/dL)/405.

### 2.4. Statistical Analysis

Statistical analyses were performed using IBM SPSS Statistics version 25 (IBM Corp., Armonk, NY, USA). No a priori sample size or power calculation was performed because of the exploratory nature of the study. The normality of continuous variables was assessed using the Shapiro–Wilk test. Normally distributed variables are presented as mean ± standard deviation and were compared using one-way analysis of variance (ANOVA); non-normally distributed variables are presented as median (interquartile range) and were compared using the Kruskal–Wallis test, with Dunn’s post hoc test and Bonferroni correction applied for pairwise comparisons. Categorical variables are presented as frequencies (percentages) and were compared using the Pearson chi-square test or Fisher’s exact test when expected cell counts were low. Because oral contraceptives and glucocorticoids may modify circulating hormone concentrations, a medication-free analysis was performed to specifically address potential treatment-related confounding. Participants receiving oral contraceptives or glucocorticoids were excluded from this analysis. In addition, metabolic parameters were reanalyzed after excluding participants receiving anti-diabetic or anti-obesity medications to assess potential treatment-related confounding. Receiver operating characteristic (ROC) curve analysis was performed to evaluate the ability of basal 17-OHP to discriminate NCAH from the combined PCOS and healthy control groups. The area under the curve (AUC) was calculated, and the optimal cutoff value was determined using the Youden index, with the corresponding sensitivity and specificity calculated.

## 3. Results

### 3.1. Study Population and Baseline Characteristics

A total of 58 women were included: 24 with PCOS, 16 with NCAH, and 18 healthy controls. There were no significant between-group differences in age (*p* = 0.069) or body mass index (*p* > 0.05). Demographic, clinical, metabolic, and hormonal characteristics of the study population are summarized in Table 1.

Within the PCOS group, phenotype classification was performed based on the combination of hyperandrogenism, ovulatory dysfunction, and available ovarian morphology data. Eight women (33.3%) met the criteria for NIH/classic phenotypes (A or B), whereas 16 (66.7%) were classified as non-NIH phenotypes, including 10 (41.7%) as phenotype C (hyperandrogenism with ovulatory preservation) and 6 (25.0%) as phenotype D. Overall, 16 of 24 women with PCOS (66.7%) were classified as having non-NIH phenotypes. Subgroup comparison of NIH (*n* = 8) versus non-NIH (*n* = 16) phenotypes showed no significant differences in AMH (*p* = 0.5), total testosterone (*p* = 0.08), androstenedione (*p* = 0.5), or metabolic parameters, with the exception of a marginally higher HbA1c in the NIH subgroup (*p* = 0.01).

### 3.2. Clinical Features

Hirsutism, assessed using the modified Ferriman–Gallwey (mFG) score, differed significantly across groups (*p* < 0.001). The mFG score was significantly higher in both the PCOS and NCAH groups than in healthy controls, with no significant difference between PCOS and NCAH. Pairwise post hoc comparisons for the mFG score are presented in Table 2. Hirsutism, defined as an mFG score ≥ 8, was more frequent in both patient groups than in controls (*p* = 0.003), without a significant difference between PCOS and NCAH (Table 1). Menstrual irregularity was also significantly more frequent in PCOS than in NCAH or controls (*p* = 0.040). Reproductive history, including the number of healthy pregnancies, parity, and abortions, did not differ across the three groups.

### 3.3. Metabolic Parameters

Metabolic profiles were largely similar across the three groups. Fasting glucose, HOMA-IR, HbA1c, HDL cholesterol, and LDL cholesterol did not differ significantly (all *p* > 0.05). Triglyceride levels differed across groups on the Kruskal–Wallis test (H = 6.473, df = 2, *p* = 0.039), with the lowest levels observed in controls; however, no pairwise comparison remained significant after Bonferroni correction. The corresponding pairwise post hoc comparisons for triglycerides are presented in Table 2. In an additional sensitivity analysis excluding the six participants receiving anti-diabetic or anti-obesity medications, fasting glucose (*p* = 0.446), HOMA-IR (*p* = 0.189), HbA1c (*p* = 0.829), HDL cholesterol (*p* = 0.874), and LDL cholesterol (*p* = 0.225) remained non-significantly different among the groups. The overall difference in triglyceride levels was attenuated and no longer reached conventional statistical significance (*p* = 0.050).

### 3.4. Hormonal Parameters

Gonadotropins (FSH and LH), estradiol, and prolactin did not differ significantly among the groups (all *p* > 0.05). In contrast, the androgen axis showed significant between-group differences. Basal 17-OHP was markedly higher in the NCAH group than in either the PCOS or control groups (*p* < 0.001); post hoc analysis confirmed that NCAH differed significantly from both other groups, whereas PCOS and controls did not differ from each other (Table 2). ROC analysis of basal 17-OHP for discriminating NCAH from the combined PCOS and healthy control groups yielded an AUC of 1.000. The optimal cutoff value was 2.40 ng/mL, providing 100% sensitivity and 100% specificity in the present cohort.

**Figure 1:**
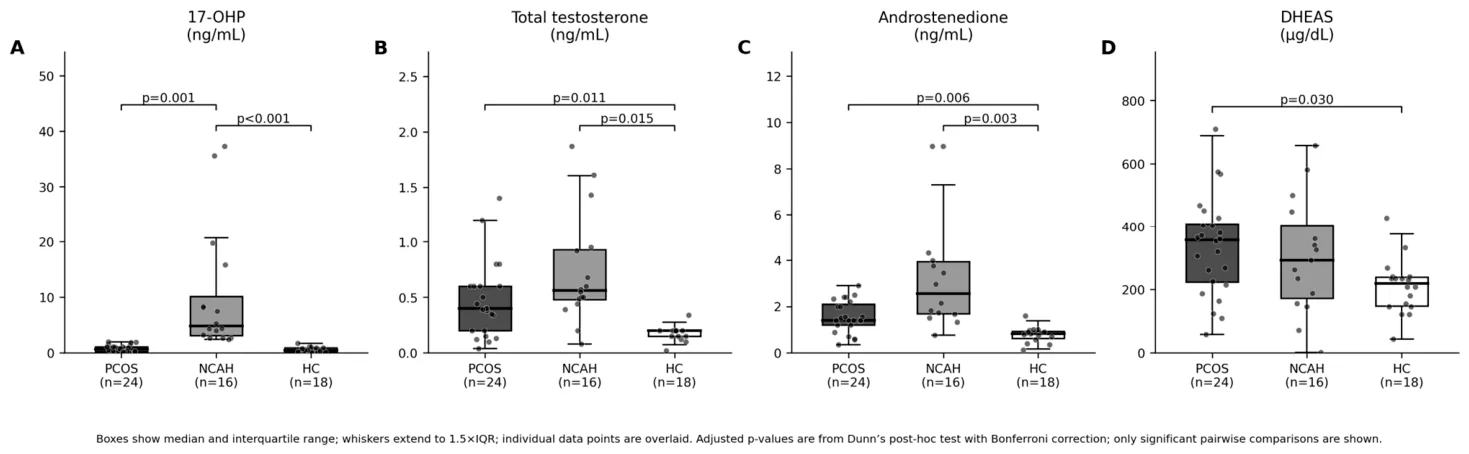
Comparison of hormonal parameters among the PCOS, NCAH, and healthy control groups: (**A**) 17-hydroxyprogesterone (17-OHP); (**B**) total testosterone; (**C**) androstenedione; and (**D**) dehydroepiandrosterone sulfate (DHEAS). Boxes show median and interquartile range; whiskers extend to 1.5 × IQR; individual data points are overlaid. Adjusted *p*-values are from Dunn’s post-hoc test with Bonferroni correction; only significant pairwise comparisons are shown.

Total testosterone and androstenedione were also significantly different across groups (both *p* < 0.001). For both parameters, the PCOS and NCAH groups had significantly higher levels than controls but did not differ from each other. DHEAS differed across groups (*p* = 0.020), with the only significant pairwise difference being between PCOS and controls; NCAH did not differ significantly from either group. In contrast, anti-Müllerian hormone did not differ among the three groups (*p* = 0.600). Pairwise post hoc comparisons for hormonal parameters with significant overall between-group differences are presented in Table 2 and Figure 1.

### 3.5. Medication-Free Analysis

Because oral contraceptives and glucocorticoids may partially modify the hormonal abnormalities under investigation, analyses were repeated after excluding all 12 participants receiving either treatment, leaving 46 untreated participants (PCOS, *n* = 18; NCAH, *n* = 10; healthy controls, *n* = 18). The principal hormonal findings remained unchanged. Significant between-group differences persisted for 17-OHP (*p* < 0.001), total testosterone (*p* < 0.001), androstenedione (*p* = 0.003), and DHEAS (*p* = 0.011). The corresponding post hoc patterns were unchanged: NCAH differed from both PCOS and controls in 17-OHP; both patient groups had higher total testosterone and androstenedione concentrations than controls without differing significantly from each other; and DHEAS differed significantly only between PCOS and controls.

### 3.6. Diagnostic Performance and ROC Curve Analysis

Receiver operating characteristic (ROC) curve analysis was performed to evaluate the discriminatory performance of basal 17-OHP for NCAH. Basal 17-OHP demonstrated complete discrimination between NCAH and the combined PCOS and healthy control groups, with an area under the curve (AUC) of 1.000 (standard error = 0.000; 95% CI, 1.000–1.000). The optimal cutoff determined by the Youden index was 2.40 ng/mL, yielding a sensitivity of 100% and a specificity of 100%.

## 4. Discussion

In this exploratory analysis of a PCOS cohort in which 66.7% of women were classified as having non-NIH Rotterdam phenotypes, women with PCOS and women with NCAH showed no significant differences across nearly the entire clinical and biochemical spectrum examined. Circulating testosterone and androstenedione, reproductive history, and the full metabolic panel showed no significant differences between the two patient groups, while both patient groups showed greater clinical hyperandrogenism, as assessed by the mFG score, and higher androgen concentrations than healthy controls. The single parameter that separated NCAH from both PCOS and controls was basal early-follicular 17-OHP. Menstrual irregularity was the only clinical feature more frequent in PCOS, and DHEAS distinguished PCOS from controls but not from NCAH. These findings suggest that, against a milder phenotypic background, the two disorders may converge almost completely at the level of clinical presentation and peripheral androgen concentrations, diverging only at the point of the enzymatic block itself.

Our androgen findings largely reproduce the pattern described in the comparative literature, with one informative exception. The overlap in hirsutism is well established: Chanukvadze et al. reported hirsutism in 80% of PCOS and 75.9% of NCAH patients with no difference in hirsutism score, and a large Portuguese cohort similarly found NCAH patients to be more hirsute than controls but indistinguishable from other hyperandrogenic groups, including PCOS [16,17]. In our cohort, this clinical overlap was also evident in the mFG score, which was higher in both patient groups than in healthy controls but did not differ between PCOS and NCAH. Androstenedione behaved as expected, rising in both disorders without discriminating between them [16,18]. The exception concerns testosterone: the literature generally reports higher testosterone in PCOS than in NCAH, reflecting ovarian theca-cell hyperresponsiveness to LH, whereas we found no difference, with numerically higher values in NCAH [14,19]. Two considerations may reconcile this. First, the reported difference is not universal; Chanukvadze et al. found no significant difference in total, calculated free, or bioavailable testosterone, indicating substantial individual-level overlap. Second, and more specific to our cohort, the ovarian contribution to testosterone excess is expected to be least pronounced in non-NIH phenotypes; consistent with this, LH and the LH-driven pattern that typically accompanies classic PCOS were not elevated in our PCOS group. Against this attenuated ovarian background, the adrenal androgen output of NCAH is sufficient to match or exceed that of PCOS. Biochemically, 21-hydroxylase deficiency promotes the accumulation of adrenal androgen precursors, particularly androstenedione, which can subsequently be converted to testosterone through 17β-hydroxysteroid dehydrogenase (17β-HSD), providing a mechanistic explanation for the relatively high testosterone concentrations observed in the NCAH group [20]. In this setting, 17-OHP retained its discriminatory value even as a basal measurement, in keeping with its established position as the principal screening test [9,21]; notably, this held true after excluding women on glucocorticoid therapy, in whom 17-OHP suppression might otherwise have attenuated the difference. Moreover, 17-OHP retained clear discriminatory value even when measured by immunoassay, a method with lower specificity than LC-MS/MS, suggesting that the separation observed is robust and would likely be at least as pronounced with mass spectrometry-based measurement. It should be acknowledged, however, that the diagnosis of NCAH had itself been based on an elevated 17-OHP response, introducing a potential incorporation bias or circular reasoning constraint; the separation of NCAH by basal 17-OHP is therefore expected by design and does not represent an independent validation of its diagnostic performance. Rather, the central finding of this study lies in the convergence of all other parameters—clinical, metabolic, and androgen—between the two groups, against which 17-OHP stands out as the sole point of divergence.

The absence of metabolic differences between our groups is consistent with, rather than contrary to, existing evidence, once cohort composition is taken into account. Direct comparative studies place PCOS above NCAH in metabolic burden, but this excess is concentrated in obese PCOS: Pall et al. found no significant metabolic differences between lean PCOS and NCAH, with the greatest dysfunction confined to the obese PCOS group, and the systematic review by Papadakis et al. concluded that the metabolic profile of NCAH resembles that of lean PCOS [9,11]. In the largest direct comparison, NCAH showed a less unfavorable profile than PCOS while remaining abnormal relative to controls [19]. Our cohort, dominated by non-NIH phenotypes with a median BMI in the overweight range and no elevation in HOMA-IR, corresponds to precisely the subgroup in which this metabolic separation disappears. Triglycerides showed an overall between-group difference, with the lowest values in controls, but no pairwise comparison survived Bonferroni correction, and this isolated finding should not be overinterpreted. The broader implication is the one already emphasized in the literature: metabolic assessment cannot be used to exclude NCAH in a woman presenting with hyperandrogenism [9,15].

Two findings warrant separate consideration because they diverge from common expectation. AMH did not differ across the three groups, including between PCOS and controls. This is best understood in light of the phenotype-dependence of AMH: a recent meta-analysis of 49 studies comprising 15,535 women demonstrated that AMH concentrations vary markedly across Rotterdam phenotypes, being highest in phenotype A (11.49 ng/mL) and progressively lower in phenotypes D (8.97), C (7.98), and B (6.25), with polycystic ovarian morphology exerting the greatest influence, followed by oligo-anovulation and hyperandrogenism [22]. Our cohort, in which 66.7% of women had non-NIH phenotypes, would therefore be expected to display AMH concentrations at the lower end of the PCOS range—as observed, with a median well below the values typically reported in phenotype A cohorts. Consistent with this, the limited direct evidence in NCAH indicates that AMH is usually within the normal range and does not reliably separate NCAH from the broader spectrum of PCOS [23]. AMH did not differ between NIH and non-NIH subgroups within our PCOS cohort (*p* = 0.5), suggesting that the low AMH concentrations observed were not attributable to phenotype composition alone. However, with only eight women in the NIH subgroup, this comparison was underpowered, and a phenotype-related difference cannot be excluded. Assay-related factors are also likely to contribute: AMH was measured with a research-use ELISA rather than an automated clinical platform, and AMH concentrations are known to vary substantially across assays, precluding direct comparison with the Beckman Coulter Gen II-converted values reported in the meta-analysis. Overall, AMH lacked discriminatory value across the entire cohort rather than only in its milder subset. DHEAS showed the converse pattern to that sometimes assumed: it was elevated in PCOS relative to controls but did not distinguish NCAH from either group. Although DHEAS is often raised in NCAH, this is neither universal nor specific. Piróg et al. found NCAH DHEAS higher than controls but not different from PCOS, and Fanta et al. reported no DHEAS elevation in any of eight NCAH patients despite androgen excess [19,24]. Adrenal androgen involvement is also common in PCOS itself [25]. DHEAS is therefore better regarded as a supportive marker of adrenal androgen contribution than as a discriminator between the two conditions.

Taken together, these findings carry a specific clinical implication for the milder end of the PCOS spectrum. A woman with hyperandrogenism, preserved or only mildly disturbed menstrual function, an unremarkable metabolic profile, and a normal AMH may appear reassuringly consistent with a mild, ovulatory PCOS phenotype, and data show no significant difference from NCAH on any parameter except 17-OHP. These findings are also relevant in the context of previous epidemiological data from Türkiye. In an unselected population of reproductive-aged Turkish women, the prevalence of PCOS was reported as 6.1% according to the NIH criteria and 19.9% according to the Rotterdam criteria [26], highlighting the substantial effect of diagnostic criteria on reported PCOS prevalence. NCAH accounts for approximately 4.2% of hyperandrogenic women worldwide, with 2.1% reported among Turkish hirsute hyperandrogenic women and up to 10% in some Mediterranean series of women presenting with hirsutism and PCOS [27,28,29]. Misclassification is not merely academic: recognition of NCAH alters fertility counseling, carries implications for genetic counseling and carrier risk, and may change management [8,30]. Our data support the position that basal early-follicular 17-OHP should be measured in all women evaluated for hyperandrogenism, irrespective of how mild or metabolically benign the presentation appears, with ACTH stimulation reserved for borderline values.

### 4.1. Limitations

This study has several limitations. The sample size was modest, particularly in the NCAH group, which limited statistical power. Consequently, the absence of significant differences between PCOS and NCAH, for example, in testosterone, androstenedione, or metabolic parameters, should be interpreted as a failure to demonstrate a difference rather than as evidence of true equivalence; larger studies may reveal differences that the present sample was underpowered to detect. Systematic follicle counts and ovarian volume measurements were not available for all participants; consequently, PCOM could not be uniformly confirmed, complete Rotterdam phenotype subclassification (A versus B and C versus D) was not feasible, and the reported phenotype distribution should be regarded as approximate rather than definitive. Although 66.7% of the PCOS group were classified as having non-NIH phenotypes, 33.3% met NIH criteria, and although subgroup comparison showed no significant differences in AMH or androgen levels between NIH and non-NIH phenotypes, this analysis was underpowered; the interpretation of our findings as reflecting a milder phenotypic background should therefore be regarded as provisional. NCAH had been diagnosed previously by ACTH-stimulated 17-OHP, whereas the present analysis used basal values; while this reflects first-line clinical practice, the discriminatory performance reported here applies to basal measurement only. AMH was measured using a research-use ELISA kit rather than an automated clinical platform, and given the well-documented inter-assay variability of AMH, the absolute concentrations reported here should not be directly compared with values from other platforms. In addition, assay-specific intra- and inter-assay coefficients of variation and functional sensitivity values for total testosterone, androstenedione, and 17-OHP could not be retrospectively verified for the measurements performed during the study period, which limits a more detailed assessment of analytical precision. Finally, the single-center design may limit generalizability, particularly given the known ethnic variation in NCAH prevalence. Although mFG scores were derived from available clinical assessments, pretreatment scoring could not be confirmed for all participants; therefore, treatment exposure may have influenced hirsutism assessment in a subset of patients. Notably, results were unchanged in sensitivity analyses excluding women receiving oral contraceptives or glucocorticoids, which argues against treatment effects as a source of bias.

### 4.2. Future Directions

Future studies should validate these findings prospectively in larger, multicenter cohorts with standardized clinical, biochemical, and ultrasonographic assessments. Recruitment of treatment-naïve participants and systematic assessment of ovarian morphology would allow a more robust evaluation of differences across PCOS phenotypes and between PCOS and NCAH. In particular, the discriminatory performance of basal early-follicular 17-OHP should be prospectively evaluated using standardized ACTH stimulation testing as the diagnostic reference. Larger studies using standardized AMH assays are also needed to determine whether AMH has discriminatory value in specific PCOS phenotypes or NCAH subgroups. Such studies may help refine practical diagnostic algorithms for women presenting with hyperandrogenism and reduce misclassification between PCOS and NCAH.

## 5. Conclusions

In this cohort, in which 66.7% of women with PCOS were classified as having non-NIH phenotypes, PCOS and NCAH showed substantial overlap across the clinical, metabolic, and androgen parameters examined, whereas basal early-follicular 17-OHP was the only parameter that consistently distinguished NCAH from both PCOS and healthy controls. However, given the modest sample size, the absence of significant differences in other parameters should not be interpreted as evidence of equivalence between PCOS and NCAH. AMH did not differ significantly among the groups and did not provide additional discriminatory value. These findings support the inclusion of basal 17-OHP in the evaluation of women presenting with hyperandrogenism, irrespective of how mild the clinical presentation or how unremarkable the metabolic and ovarian-reserve profile appears. More broadly, accurate differentiation between PCOS and NCAH is important for appropriate management, fertility counseling, and genetic counseling, as well as for reducing diagnostic misclassification in women with overlapping hyperandrogenic phenotypes.

## Figures and Tables

**Table 1:** Demographic, clinical, metabolic, and hormonal characteristics of the study population.

Variable	PCOS (*n* = 24)	NCAH (*n* = 16)	HC (*n* = 18)	*p*-Value
Demographic and anthropometric	Demographic and anthropometric	Demographic and anthropometric	Demographic and anthropometric	Demographic and anthropometric
Age (years)	25.5 ± 4.7	29.6 ± 8.6	29.7 ± 6.9	0.069
BMI ^a^ (kg/m^2^)	28.2 [23.7–32.7]	26.9 [23.1–33.4]	26.4 [21.5–33.1]	0.500
Clinical features	Clinical features	Clinical features	Clinical features	Clinical features
Hirsutism (mFG score ≥ 8), *n* (%)	16 (66.7)	12 (75)	4 (22.2)	**0.003**
mFG score ^a^	9.5 [4.5–12.8]	12 [5.3–14.8]	3.0 [2,3,4,5]	**<0.001**
Menstrual irregularity, *n* (%)	13 (54.2)	5 (31.3)	3 (16.7)	**0.040**
Reproductive history	Reproductive history	Reproductive history	Reproductive history	Reproductive history
Healthy pregnancies ^a^	0 [0–1]	0 [0–2]	0 [0–1]	0.400
Parity ^a^	0 [0–1]	0 [0–2]	0 [0–1]	0.500
Abortions ^a^	0 [0–0]	0 [0–0]	0 [0–0]	0.700
Metabolic parameters	Metabolic parameters	Metabolic parameters	Metabolic parameters	Metabolic parameters
Fasting glucose ^a^ (mg/dL)	92 [84–97]	92.5 [88.5–101.8]	92 [82.8–102.5]	0.800
HOMA-IR^a^	2.87 [2–3.86]	4.53 [2.25–6.17]	2.21 [1.09–4.86]	0.400
HbA1ca (%)	5.4 [5.1–5.7]	5.5 [5.1–5.9]	5.4 [5.2–5.5]	0.600
Triglycerides ^a^ (mg/dL)	138 [89–173]	138.5 [84.5–198.8]	89 [57.3–117.8]	**0.039**
HDL cholesterol ^a^ (mg/dL)	51.5 [42.3–65]	60 [44–66.3]	60 [52–65]	0.600
LDL cholesterol ^a^ (mg/dL)	105 [90–120]	104 [91.8–115]	82.5 [77–112.8]	0.100
Hormonal parameters	Hormonal parameters	Hormonal parameters	Hormonal parameters	Hormonal parameters
FSHa (IU/L)	6.0 [5.2–6.6]	6.29 [4.4–7.6]	6.1 [5.4–7.5]	0.600
LHa (IU/L)	6.4 [4.7–10.7]	7.28 [4.4–11]	5.9 [4.8–7.4]	0.700
Estradiola (ng/L)	41 [34–47]	40.5 [28.5–63.1]	38.5 [28.5–48.8]	0.900
Total testosterone ^a^ (ng/mL)	0.4 [0.2–0.6]	0.59 [0.45–0.94]	0.2 [0.15–0.2]	**<0.001**
Androstenedione ^a^ (ng/mL)	1.4 [1.2–2.2]	2.55 [1.64–4.07]	0.87 [0.56–1]	**<0.001**
DHEASa (µg/dL)	356.5 [217–422]	292 [156–448]	219 [146–240]	**0.020**
17-OHPa (ng/mL)	0.67 [0.2–1.1]	4.83 [2.83–13.9]	0.4 [0.17–0.85]	**<0.001**
Prolactina (ng/mL)	15 [10.2–27.3]	22.05 [14.6–26.3]	16.2 [10.8–21.7]	0.300
AMHa (ng/mL)	3.27 [3.02–4.13]	3.13 [2.87–3.54]	3.34 [2.93–5.37]	0.600

^a^ Values are presented as median [interquartile range]; all other continuous variables as mean ± standard deviation. Categorical variables are presented as *n* (%). Between-group comparisons were performed using one-way ANOVA (age), the Kruskal–Wallis test (other continuous variables), or the Pearson chi-square/Fisher’s exact test (categorical variables). Significant *p*-values (<0.05) are shown in bold. Basal early-follicular 17-OHP is reported. Abbreviations: AMH, anti-Müllerian hormone; BMI, body mass index; DHEAS, dehydroepiandrosterone sulfate; FSH, follicle-stimulating hormone; HC, healthy controls; HDL, high-density lipoprotein; HOMA-IR, homeostatic model assessment of insulin resistance; LDL, low-density lipoprotein; LH, luteinizing hormone; mFG, modified Ferriman–Gallwey; NCAH, nonclassic congenital adrenal hyperplasia; 17-OHP, 17-hydroxyprogesterone; PCOS, polycystic ovary syndrome.

**Table 2:** Bonferroni-adjusted pairwise (post hoc) comparisons for parameters with a significant overall difference.

	PCOS vs. NCAH	PCOS vs. HC	NCAH vs. HC	*p*-Value
17-OHP	**<0.001**	0.900	**<0.001**	**<0.001**
Total testosterone	0.200	**0.030**	**<0.001**	**<0.001**
Androstenedione	0.070	**0.010**	**<0.001**	**<0.001**
DHEAS	1	**0.020**	0.200	**0.020**
Triglycerides	1	0.080	0.090	**0.039**
mFG	1	**<0.001**	**<0.001**	**<0.001**

Values are Bonferroni-adjusted *p*-values from Dunn’s post hoc test following a significant Kruskal–Wallis test. The overall *p*-value is from the Kruskal–Wallis test. Significant values (<0.05) are shown in bold. Abbreviations: DHEAS, dehydroepiandrosterone sulfate; HC, healthy controls; NCAH, nonclassic congenital adrenal hyperplasia; 17-OHP, 17-hydroxyprogesterone; PCOS, polycystic ovary syndrome; mFG, modified Ferriman–Gallwey.

## Data Availability

The data presented in this study are available from the corresponding author upon reasonable request. The data are not publicly available due to privacy and ethical restrictions.
